# Kikuchi-Fujimoto disease as the initial manifestation of systemic lupus erythematosus complicated with macrophage activation syndrome: two case reports and a review of literature

**DOI:** 10.1186/s12887-022-03703-6

**Published:** 2022-11-22

**Authors:** Chenxi Liu, Yingying Jin, Hua Huang, Fei Ding, Zhen Yang, Xuemei Xu, Shengfang Bao, Jing Ma, Yanliang Jin

**Affiliations:** 1 Department of Rheumatology and Immunology, Shanghai Children’s Medical Center, Shanghai Jiao Tong University School of Medicine, Shanghai, 200127 China; 2 Department of Pathology, Shanghai Children’s Medical Center, Shanghai Jiao Tong University School of Medicine, Shanghai, 200127 China

**Keywords:** Kikuchi-Fujimoto disease, Systemic lupus erythematosus, Macrophage activation syndrome, Case report

## Abstract

**Background:**

Kikuchi-Fujimoto disease (KFD) is a self-limiting and benign disease characterized by cervical lymphadenopathy and fever. Although KFD should be made differentially diagnosed from infectious, autoimmune, and malignant diseases, it sometimes occurs in patients with systemic lupus erythematosus (SLE) and can be complicated with macrophage activation syndrome (MAS). However, it is rare that KFD is the initial manifestation of SLE and to be complicated with MAS.

**Case presentation:**

A 9.6-year-old girl presented with high-grade fever, double-side cervical lymphadenopathy with mild pain of one week, leukopenia, alopecia, and rash on the cheek. During hospitalization, laboratory investigations showed positive antinuclear antibody (ANA), low complement 3 (C3), and low complement 4 (C4). Imaging investigations showed pleural and pericardial effusion. A 10.3-year-old girl presented with intermittent high-grade fever, double-sided cervical lymphadenopathy with obvious pain of 1-month duration, and discoid lesion on the cheek. During hospitalization, laboratory investigations showed positive ANA, leukopenia, thrombocytopenia, anemia with positive Coombs’ test, low C3, and positive Smith antibodies. Both cases were diagnosed with KFD using lymph node biopsy, simultaneously fulfilling the diagnostic criteria of SLE. Subsequently, the two girls became complicated with MAS, followed by interstitial lung disease and neuropsychiatric lupus, respectively. Both patients benefited from high-dose methylprednisolone pulse therapy combined with intravenous cyclophosphamide.

**Conclusions:**

More attention should be paid to differential diagnosis, especially SLE, in children diagnosed with KFD. In addition, children with SLE who presented with KFD as the initial manifestation seem to have a higher risk of developing MAS and experiencing organ involvement.

## Background

Kikuchi-Fujimoto disease (KFD), initially reported in Japan by pathologists Kikuchi and Fujimoto in 1972 [1], is a self-limiting and benign disease characterized by fever and cervical lymphadenopathy. Although KFD has been reported worldwide, it has a higher prevalence in young adult female Asians [2]. However, KFD is rare and may have a male predominance in pediatric patients [3–5]. As a systemic disease with symptoms of generalized lymphadenopathy, upper respiratory tract infection, night sweats, chills, arthralgia, rash, weight loss, and neurological involvement, its etiology remains unknown. However, there is a view suggests that KFD may be related to infectious and autoimmune processes, emphasizing that it may coexist or present mimicking various conditions, such as viral infections (e.g., Epstein-Barr virus [EBV] and cytomegalovirus [CMV]), autoimmune diseases (e.g., systemic lupus erythematosus [SLE]), and malignant diseases (e.g., lymphoma). Several studies have shown that whether in adults or pediatric patients, KFD may have a certain association with SLE that can be diagnosed in its different stages; however, the incidence is low [2–6]. Macrophage activation syndrome (MAS), a hemophagocytic lymphohistiocytosis secondary to rheumatic disease, is a life-threatening condition. It is rare that KFD is the initial manifestation of SLE and to be complicated with MAS.

Herein, we reported two Chinese girls who presented with fever and cervical lymphadenopathy. Their lymph node biopsy was positive for KFD after hospitalization; simultaneously, they also fulfilled the diagnostic criteria of SLE. Subsequently, their condition was complicated with MAS.

## Case presentation 1

**Fig. 1:**
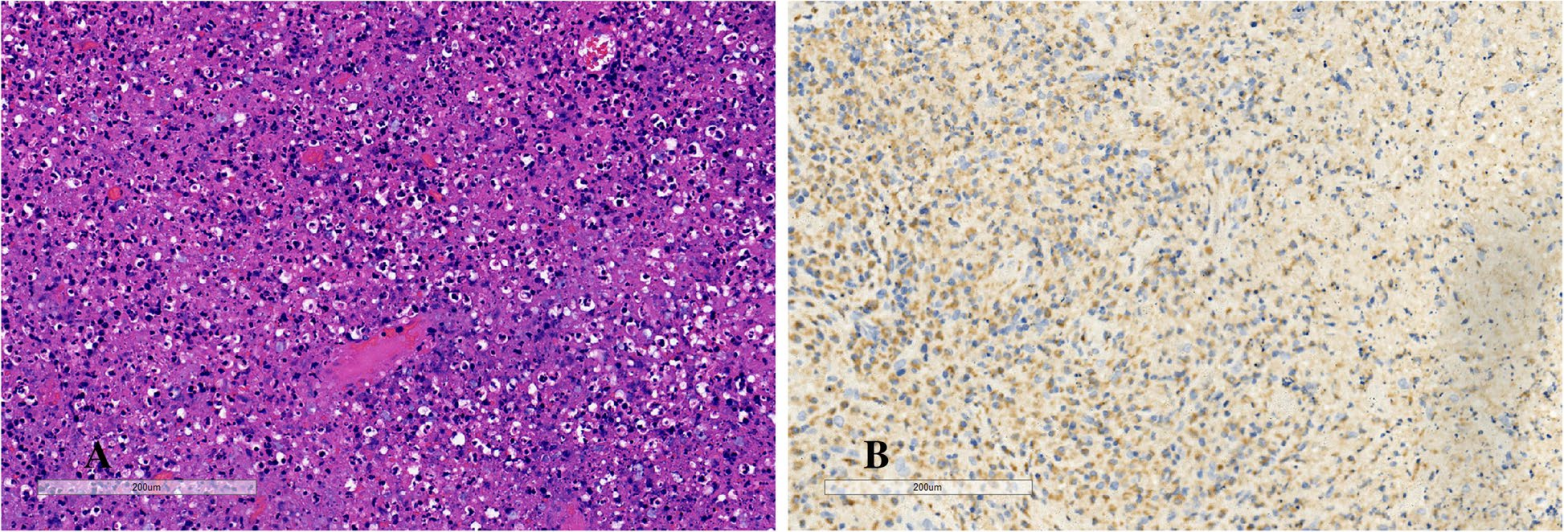
**A** Case 1, high power view of an area of lymphocytic proliferation, with large lymphocytes, histiocytic cells and occasional nuclear debris. **B** Immunohistochemistry of the patient’s cervical lymph nodes obtained from biopsy. CD68( +) histiocytes surround the necrotic areas

A 9.6-year-old previously healthy schoolgirl presented with high-grade fever, double-side cervical lymphadenopathy with a mild pain of 1 week duration, and leukopenia (white blood cell count [WBC] 3.65 × 10^9^/L, normal 4–12 × 10^9^/L). She also had alopecia, rash on the cheek, headache, throat discomfort, intermittent cough, and alopecia. She denied weight loss, arthralgia or exhaustion and physical examination showed no hepatomegaly or splenomegaly. There were no night sweats, hemoptysis or contact history of tuberculosis (TB). There was also no significant high-risk sexual, travel, any past medical, or family illnesses histories. Fever persisted without significantly increasing of C-reactive protein (CRP) after 5 days of intravenous treatment with azithromycin and cephalosporins prescribed by other hospitals. Subsequently, she was transferred to our hospital, and on day 11th of fever, she underwent a right-sided cervical lymph node biopsy. The postoperative pathology showed lymphocytic proliferation with large lymphocytes, histiocytic cells, and occasional nuclear debris without hematoxylin bodies and neutrophils (Fig. 1A). Immunohistochemistry was positive for lysozyme (Fig. 1B). The diagnosis of KFD was made based on these pathological manifestations (Table 1).

**Table 1:** Characteristics and manifestations that conform to the diagnostic criteria

**Patients**	**Age/Sex**	**Features consistent with the diagnosis of KFD**	**Satisfied diagnostic criteria for SLE according to EULAR/ACR2019 **[7]	**Satisfied diagnostic criteria for MAS **[8]
Case 1	9-year- old/ girl	Clinical symptoms: Fever, lymphadenopathy Laboratory investigations: leukopenia Pathology: KFD Immunohistochemistry: EBER(-), ALK1(-),CD10(-), CD20(-), CD21(-), CD30(-), CD34(-), CD38(-), CD4( +), CD8( +), CK(-), KP-1( +), LCA( +), Lys( +), MUM1(-), PAX5(-), PGM1( +)	ANA at a titer of 1:320 Fever > 38.3℃ (score 2) 2. Leukopenia, thrombocytopenia (score 4) alopecia, rash (score 4) Pleural or pericardial effusion (score 5) 4. Low C3 and low C4 (score 4) Total score: 19	Clinical criteria: 1. Fever with T > 38℃ 2. Hepatomegaly and splenomegaly 3. Central nervous system symptoms (dim) Laboratory criteria: Thrombocytopenia (minimum number: 3 × 10^9/L), anemia (minimum number: 56 × 10^9/L) AST (maximum number: 652 U/L) LDH (maximum number: > 8600 U/L) SF (maximum number: > 6000 ug/L) FIB (minimum number: 0.62 g/L)
Case 2	10-year- old/ girl	Clinical symptoms: Fever, lymphadenopathy Laboratory investigations: leukopenia Pathology: Necrotizing lymphadenitis Immunohistochemistry: LCA( +), CD20( +),PAX5( +), CD3( +), CD2( +), CD30( +), EMA( +), CD10( +), CD15( +), CD34( +), TdT(-), Ki-67( +), CD21( +), CD38( +), ALK(-), CD68( +)	ANA at a titer of 1:1000 Fever (score 2) Leukopenia, thrombocytopenia, Coombs’ test positive (score 4) Subacute discoid lupus (score 4) Low C3 (score 3) Anti-Sm antibody positive (score 6) Total score: 19	Clinical criteria: 1. Fever with T > 38℃ Laboratory criteria: Thrombocytopenia (minimum number: 101 × 10^9/L), leukopenia (minimum number: 2.15 × 10^9/L) 3. AST (maximum number: 59 U/L) 4. LDH (maximum number: 3156 U/L) 5. SF (maximum number: 1420.6 ug/L)

*Abbreviations: KFD* Kikuchi-Fujimoto disease, *SLE* systemic lupus erythematosus, *MAS* Macrophage activation syndrome, *ANA* antinuclear antibody, *AST* aspartate aminotransferase, *LDH* lactate dehydrogenase, *SF* serum ferritin, *anti-Sm antibody* Smith antibodies, *C3* complement 3, *C4* complement 4, *EULAR/ACR2019* the European League Against Rheumatism and the American College of Rheumatology 2019, *FIB* fibrinogen

Other examinations were performed for the differential diagnosis and condition assessment, simultaneously. Laboratory investigation showed positive antinuclear antibody (ANA) was with a titer of 1:320, the anti-ribosome antibodies test (anti-rRNP) was positive, and complement 3 (C3) and complement 4 (C4) levels were low (C3 0.77 g/L, normal 0.9–1.8 g/L; C4 0.04 g/L, normal 0.1–0.4 g/L, respectively); however, the complement total activity (CH50) was normal (36 U/ml, normal 23–46 U/ml). The result of the TB interferon-gamma release assay (T-sport), the serology for EBV, CMV, and human immunodeficiency virus (HIV), and the test for hepatitis B surface antigen and hepatitis C antibody were negative. The bone marrow aspirations showed that no obvious abnormality in three-lineage hyperplasia, and no immature lymphocytes were found. Anti-cardiolipin antibody was negative; urinary protein was < 0.5 g/24 h. Blood chemistry, including aspartate aminotransferase (AST), alanine aminotransferase (ALT), lactate dehydrogenase (LDH), and serum ferritin (SF), were normal. Inflammatory biomarkers revealed CRP of 9 mg/L (normal < 0.8 mg/L) and erythrocyte sedimentation rate (ESR) of 30 mm/h (normal 0–20 mm/h). Heart ultrasound revealed a small amount of pericardial effusion, and chest high-resolution computed tomography (HRCT) revealed bilateral pleural effusion. The diagnosis of SLE was made according to the European League Against Rheumatism and the American College of Rheumatology 2019 (EULAR/ACR2019) diagnostic criteria [7] with a score of 19 (fever, leukopenia, alopecia, rash, pleural or pericardial effusion, and low C3 and C4) (Table 1).

During this period, the fever persisted for nearly 3 weeks until intravenous methylprednisolone 2 mg/(kg·d) was administered for 1 week. At almost the same time as the diagnosis of SLE, 1 day after the temperature returned to normal, the patient started experiencing eyelid edema, mental dullness, and increased rash on the face. Further laboratory evaluation showed that the ESR levels returned to normal, with thrombocytopenia (3 × 10^9^/L, normal 100–550 × 10^9^/L), elevated SF (> 6000 ug/L, normal 4.6–204 ng/mL), elevated LDH (> 8600 U/L, normal 313–618 U/L), elevated AST (652 U/L, normal 15–46 U/L), hypertriglyceridemia (3.35 mmo/L, normal < 2.26 mmol/L), and hypofibrinogenemia (0.62 g/L, normal 1.5–4.0 g/L). The ultrasonic abdominal examination revealed hepatomegaly and splenomegaly. Bone marrow puncture was performed again, and the results indicated megakaryocytopenia. These findings indicated MAS according to the diagnostic criteria of SLE-MAS, a preliminary guideline based on a multinational multicenter study (Table 1) [8]. She was treated with intravenous methylprednisolone pulse therapy (15 mg/[kg·d]) for 3 days combined with intravenous immunoglobulin (IVIG), 1 g/[kg·d] for 2 days. Elevated ALT, hypertriglyceridemia, and hypofibrinogenemia improved significantly. In addition, SF and LDH levels decreased gradually were unremarkable. However, refractory thrombocytopenia (fluctuates between 10–40 × 10^9^/L) persisted for almost 1 month and did not improve until two doses of rituximab (375 mg/m^2^) were administered at 2-weeks intervals. Prednisolone, combined with hydroxychloroquine (HCQ) and cyclosporine A (CsA), was administered for SLE.

**Fig. 2:**
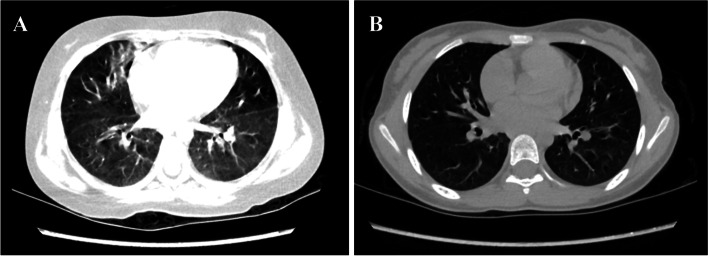
Comparison of chest HRCT images before (**A**) and after (**B**) treatment of CYC. **A** Diffused interstitial changes in both lungs. The brightness of the two lungs is uneven, extensive ground glass shadows, fiber strips can be seen. **B** The uneven brightness of both lungs improved, fiber strips and patchy shadows reduced compared with before

Three months after the diagnosis of SLE and MAS, SF and LDH were still high. The patient developed sudden tachypnea, inability to lie down, and accompanied with increased heart rate, and decreased oxygen saturation. HRCT was repeated and revealed interstitial lung disease (ILD) (Fig. 2A). The examination of lung function showed a moderately decreased diffusion capacity of the lung for carbon monoxide (DLCO) with a value of 47.8%. Under this condition, CsA was changed to intravenous cyclophosphamide ([CYC] 0.75 g/m^2^/month × 7 doses). SF and LDH returned to normal, and DLCO improved to 76.9% after all seven doses of CYC. During the latest follow-up, the patient had no disease activity of SLE, HRCT had improved significantly compared with when ILD was diagnosed (Fig. 2B), and DLCO had already returned to normal.

## Case presentation 2

**Fig. 3:**
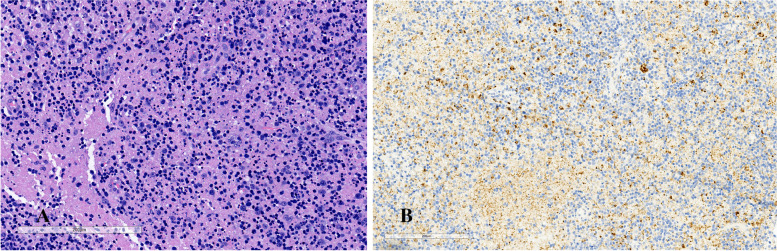
**A** Case 2, the stains of the patient’s cervical lymph nodes showing the characteristic features of histiocytic necrotizing lymphadenitis. Histiocytic infiltrate with karyorrhectic debris and large lymphocytes can be seen in this necrotic area. **B** Immunohistochemistry of the patient’s cervical lymph nodes obtained from biopsy. Lys ( +) histiocytes surround the necrotic areas

A 10.3-year-old previously healthy schoolgirl presented with intermittent high-grade fever and double-sided cervical lymphadenopathy with obvious pain for 1 month duration. Antibiotics, including amoxicillin, cefoperazone, and azithromycin, and low-dose methylprednisolone anti-inflammatory treatments in local hospitals, were not effective. The patient had no night sweats, hemoptysis, or contact history of TB. She denied weight loss, arthralgia, exhaustion, or conjunctival congestion. There was no significant high-risk sexual, travel, or past medical or family illness history. Physical examination revealed a discoid lesion on her face and generalized lymphadenopathies, including cervical and axillary. A cervical lymph node biopsy was performed on the 32nd day of fever. The postoperative pathology showed patchy necrotic areas in the paracortex made up of karyorrhectic, necrotic cell debris, and large lymphocytes without hematoxylin bodies and neutrophils (Fig. 3A). Immunohistochemistry showed CD68 was positive (Fig. 3B). The diagnosis of KFD was made based on these pathological manifestations (Table 1).

Meanwhile, laboratory investigation showed leukopenia (2.35 × 10^9^/L), anemia (99 g/L), and thrombocytopenia (101 × 10^9^/L). The ANA titer was 1:1000, and the serum C3 was 0.71 g/L (normal 0.9–1.8 g/L). Coombs’ test, Smith antibodies (anti-Sm antibody), and U1-nuclear ribonucleoprotein particle antibodies (anti-U1-nRNP) were positive. Anti-cardiolipin antibody was negative, urinary protein was < 0.5 g/24 h, and the serum C4 and CH50 were normal (0.21 g/L, normal 0.1–0.4 g/L; 40 U/ml, normal 23–46 U/ml, respectively). Echocardiography and HRCT revealed no pericardial or pleural effusion. Bone marrow puncture suggested low erythroid and megakaryocyte proliferation but with no immature lymphocytes. The results of the serology for EBV, CMV, and HIV and the test for hepatitis B surface antigen and hepatitis C antibody, and T-spot were all negative. The diagnosis of SLE was made according to EULAR/ACR2019 diagnostic criteria [7] with a score of 19 (fever, discoid lesion, leukopenia, thrombocytopenia, Coombs’ test positive, low C3, and anti-Sm antibody positive) (Table 1). Simultaneously, other laboratory investigations showed an increasing tendency of AST, LDH, SF, and triglycerides (TG), with the maximum value of 59 U/L (normal 15–46 U/L), 3156 U/L (normal 313–618 U/L), 1420.6 ng/mL (normal 4.6–204 ng/mL), and 7.03 mmol/L (normal < 2.26 mmol/L), respectively, and a decreasing tendency of fibrinogen (FIB) with the minimum value of 1.66 g/L (normal 1.5–4.0 g/L). MAS was also diagnosed according to the diagnostic criteria of SLE-MAS (Table 1) [8]. The temperature and related indicators, including AST, LDH, SF, TG, and FIB, gradually returned to normal after intravenous methylprednisolone pulse therapy of 15 mg/(kg·d) for 3 days, followed by sequential oral prednisone 2 mg/(kg·d). Furthermore, HCQ, CsA, and belimumab were administered for SLE.

**Fig. 4:**
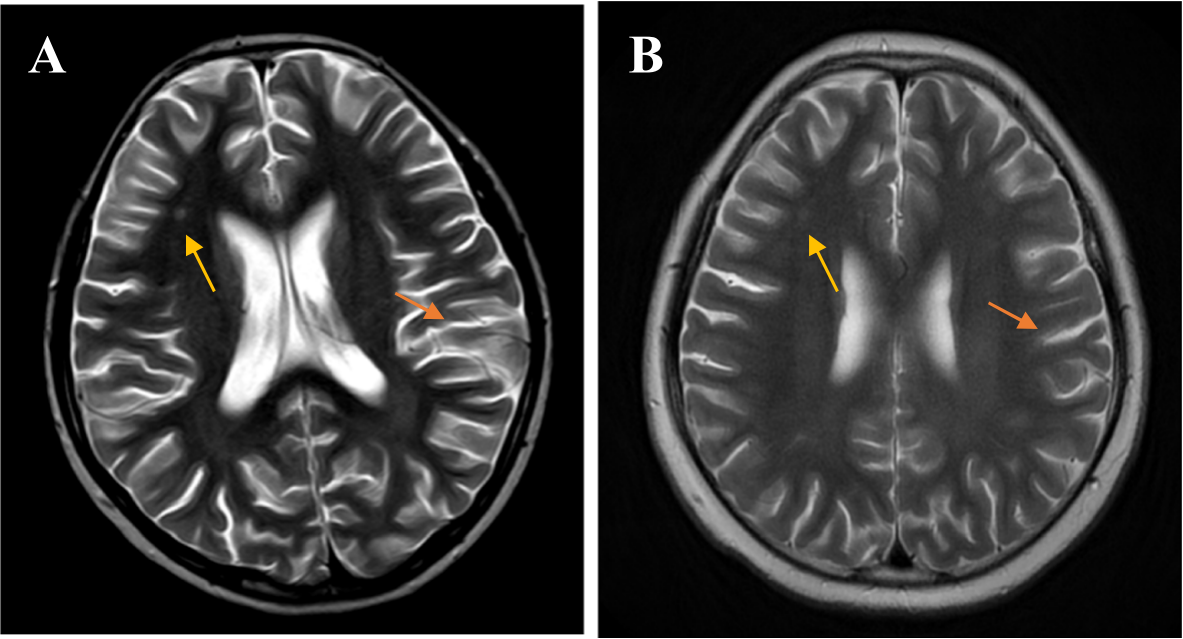
Comparison of T2-weighted head magnetic resonance imaging (MRI) before (**A**) and after (**B**) treatment of CYC. **A** A high-intensity dot zone in the right frontal white matter and high-intensity flake zone in the left temporal lobe can be seen. **B** There is no significant change of the high-intensity dot zone in the right frontal white matter and improvement of the high-intensity flake zone in left temporal lobe compared with before

However, the patient experienced hand tremors 1 month after the diagnosis of SLE and MAS. Video electroencephalogram (VEEG) was abnormal with slow wave activity in the occipital area; T2-weighted magnetic resonance imaging (MRI) of the head was also abnormal, with a high-intensity dot zone in the right frontal white matter and high-intensity flake zone in the left temporal lobe (Fig. 4A). The diagnosis of neuropsychiatric lupus was made based on the manifestations mentioned above. CsA was changed to intravenous CYC (0.75 g/m^2^/month × 7 doses), and belimumab was discontinued. During the latest follow-up, the patient had completed all seven doses of CYC with no disease activity of SLE. The repeated VEEG was normal. T2-weighted head MRI showed no significant change in the high-intensity dot zone in the right frontal white matter and improvement of the high-intensity flake zone in the left temporal lobe compared with when neuropsychiatric lupus was diagnosed (Fig. 4B).

## Discussion and conclusions

KFD, an uncommon benign disease of necrotizing histiocytic lymphadenitis, usually occurs in young East Asian females, with a male/female ratio of 1:1.26–4 in three adult cohorts [9–11]. However, most studies found that KFD in children has a male predominance, with a male/female ratio of 1.13–2.25:1 [3–5], except for two studies based on Korean children [12, 13], of which male/female ratios were 1:1 and 1:1.32, respectively. Some studies have reported that the median age of onset for children with KFD is between 8.1 and 13.2 years [3, 4, 13]. We also reviewed all 47 pediatric patients with KFD diagnosed in our hospital between April 2014 and July 2021. Among them, 31 were boys, and 16 were girls, with a male/female ratio of 1.9:1 and a median age of 10 years.

KFD may be associated with the proliferation of CD8^+^ T lymphocytes induced by autoimmunity, infection, and other factors. It may also be related to the participation of interferon-gamma and interleukin 6; however, the exact pathogenesis is unclear [14]. In clinical manifestations, the most common symptoms of KFD were lymphadenopathy, fever, rash, fatigue, or arthritis and hepatomegaly or splenomegaly [2]; various skin manifestations, including facial erythema, leukocytoclastic vasculitis, alopecia, and oral ulcerations, have also been described in KFD patients [15], which lack specificity compared with many chronic conditions, including SLE. An early study carried out by Imamura M et al. hypothesized that KFD might represent a self-limited, SLE-like autoimmune state due to various infectious agents, indicating that KFD and SLE might have a certain relationship in the occurrence mechanism. The two may also need to be carefully differentiated in diagnosis [16]. Some studies reported that the rate of KFD with or developed to SLE ranges from 1.3% to 7% [5, 12, 13, 17]. Moreover, there have also been reports of SLE diagnosed before (18%), simultaneously (51%), and after KFD (31%) [18]. Among the 47 patients with KFD in our hospital, only the two girls reported in this article had concurrent SLE and KFD as the initial manifestation. Subsequently, they developed into MAS, with the incidence of KFD with an SLE of 4.3%. According to reports, 12%–26% of patients with SLE experience lymphadenopathy at some point during the clinical stage and treatment [19–21]. Lymph node involvement is usually cervical, localized, and moderately enlarged, with a diameter of 1–2 cm in KFD, whereas lymph nodes are usually soft, mobile, generalized, and of varied size in SLE [22–24]. Lymph nodes biopsy is the gold standard for differential diagnosis. Patchy paracortical necrosis, karyorrhexis, and scarce plasma cells are the characteristics of KFD in histological findings [9]. The immunohistochemical analysis of KFD is positive for myeloperoxidase, lysozyme, CD68, and CD163. However, the presence of hematoxylin bodies, an abundance of plasma cells, the Azzopardi phenomenon (hematoxylin staining nuclear material), and sparse CD8^+^ T cells indicate SLE lymphadenitis [25]. The lymph node biopsy of our patients revealed paracortical necrosis without hematoxylin bodies, and immunohistochemistry showed lysozyme and CD68 were positive, which is compatible with KFD. In laboratory indicators, most patients with KFD have normal laboratory findings; however, some may have leukopenia (especially granulocytopenia; 20–58% of cases), leukocytosis (2–5% of cases), anemia, elevated ESR, elevated CRP, elevated LDH, elevated AST and ALT, and atypical lymphocytes in the peripheral blood, which need to be distinguished from other diseases [25]. Moreover, ANA is positive in 30% of patients with KFD, whereas anti-extractable nuclear antigen antibodies are generally negative [22]. Sopeña B et al. also found that patients with KFD-SLE had a higher probability of experiencing leukopenia, pancytopenia, anti-Ro/SSA antibodies, and positive anticardiolipin antibody immunoglobulin G than those with SLE alone [18]. Therefore, children diagnosed with KFD should be routinely tested for autoantibodies to identify the high-risk group for SLE occurrence timely.

Because KFD is related to rheumatic disease, hemophagocytic lymphohistiocytosis associated with KFD was regarded as KFD-MAS. It has been reported that the incidence of KFD-MAS was 30.8%, with more frequent glucocorticoid treatment and longer hospital stays [26]. Moreover, it is worth noting that MAS is also a complication of SLE, which often occurs in the early stage of onset, with an incidence rate of 0.9%–4.6% [27]. However, it is rare for all to occur simultaneously in one patient. There were only two reports of KFD disease associated with SLE and MAS [28, 29], except for the two cases we reported in this article. Among them is a 17-year old boy who experienced KFD-MAS at first and developed SLE 6 weeks later with no follow-up condition described after SLE [28]. The other is a 50-year-old man who experienced KFD, SLE, MAS, and neuropsychiatric lupus almost simultaneously but died due to severe infection [29]. This article reported two pediatric patients’ disease courses and detailed follow-up outcomes. It is difficult to distinguish between MAS and SLE flare since the two conditions have similar characteristics, including blood cytopenia, skin rash, fever, lymphadenopathy, splenomegaly, and neurological symptoms. The overlapping clinical manifestations may impede the identification of early MAS and result in the delay of the most appropriate treatment. Therefore, attention should be given to the occurrence of MAS in patients with SLE with unexplained fever and cytopenia, accompanied by significantly increased SF levels. SF may be a remarkable index because it increases when MAS occurs and decreases rapidly after controlling the disease [8].

Furthermore, we reported the prognosis of two patients, one with ILD and the other with neuropsychiatric lupus. Combined with the fatal case reported by other authors mentioned above, these suggested that patients with SLE with KFD as the initial manifestation may experience a more severe cytokine storm and organ involvement. Intravenous methylprednisolone pulse therapy is considered the first-line treatment [30]; however, there has been no conclusion on which immunosuppressive agent is preferable for MAS in SLE. Several studies found that CYC and CsA had a similar effect in SLE-MAS [31–33]; Kumakura et al. found CYC was beneficial in comparison with CsA [34]. Gavand et al. found CYC or etoposide should be used for uncontrolled or severe forms in SLE-MAS [30]. In addition, a review of the latest follow-up outcome of the two patients in our report showed that both benefited from CYC, and organ involvement was controlled. These outcomes show that the treatment of patients with KFD as the initial manifestation, developing into SLE and MAS, should be more aggressive, particularly in choosing immunosuppressive agents.

In conclusion, although SLE with KFD as the initial manifestation is rare, more attention should be paid to it as it may aggravate during the treatment, be more prone to developing into MAS, and can lead to the involvement of important organs. High-dose methylprednisolone pulse therapy combined with CYC may effectively control the disease and improve prognosis.

## Data Availability

The datasets used and/or analyzed during the current study are available from the corresponding author upon reasonable request.

## Abbreviations

KFD: Kikuchi-Fujimoto disease
SLE: Systemic lupus erythematosus
MAS: Macrophage activation syndrome
EBV: Epstein-Barr virus
CMV: Cytomegalovirus
ESR: Erythrocyte sedimentation rate
CRP: C reactive protein
WBC: White blood cell
TB: Tuberculosis
T-sport: TB interferon gamma release assay
HIV: Human immunodeficiency virus
ANA: Antinuclear antibody
anti-rRNP: Anti-ribosome antibodies
AST: Aspartate aminotransferase
ALT: Alanine aminotransferase
LDH: Lactate dehydrogenase
SF: Serum ferritin
Hb: Hemoglobin
PLT: Platelet
anti-Sm antibody: Smith antibodies
anti-U1-nRNP: U1-nuclear ribonucleoprotein particle antibodies
C3: Complement 3
C4: Complement 4
CH50: Complement total activity
IVIG: Intravenous immunoglobulin
EULAR/ACR2019: The European League Against Rheumatism and the American College of Rheumatology 2019
HCQ: Hydroxychloroquine
CYC: Cyclophosphamide
CsA: Cyclosporine A
MRI: Magnetic resonance imaging
HRCT: High-resolution computed tomography
DLCO: Diffusion capacity of the lung for carbon monoxide
ILD: Interstitial lung disease
FIB: Fibrinogen
